# A Serious Cause of Panic Attack

**DOI:** 10.1155/2012/393275

**Published:** 2012-06-12

**Authors:** Michael O'Connell, Aaron Bernard

**Affiliations:** Department of Emergency Medicine, The Ohio State University Medical Center, 750 Prior Hall, 376 W 10th Avenue, Columbus, OH 43210, USA

## Abstract

We report on a case of a patient with atrial fibrillation in the setting of Wolff-Parkinson-White syndrome. The patient underwent synchronized electrical cardioversion, typically considered safe and effective, which resulted in a dangerous complication for the patient (degeneration into ventricular fibrillation). Discussion of common rhythm disturbances in WPW and management strategies are reviewed.

## 1. Introduction

A 23-year-old male presented to the ED with a chief complaint of “panic attack.” He reports feeling that his heart was racing for the last hour. He also reports mild shortness of breath. He denies chest pain or feeling lightheaded. He denied anxiety but reported that he had been to another ED a few weeks prior with a similar episode and was told that it was a “panic attack.”

He denied past medical or surgical history, allergies, or medications. He reports social alcohol use and denies tobacco and illicit drug use.

Triage vital signs, BP 120/70 RR 18 Pox 99% HR, are not obtained.

**Figure 1:**
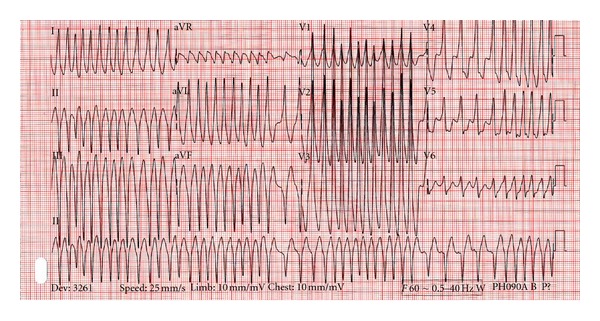
EKG showing atrial fibrillation with rapid ventricular response in the setting of Wolff-Parkinson-White disease.

On physical exam the patient was well appearing. His cardiovascular exam demonstrated irregular rhythm with no murmurs. Peripheral pulses were irregular and bradycardic. An EKG was performed which revealed the following (see Figure 1).

The patient was placed on a cardiac monitor, IV access was obtained, and a plan was made to cardiovert the patient due to the unstable nature of the rhythm. Use of IV procainamide was discussed but was not readily available. The patient was given intravenous fentanyl and midazolam, and synchronized cardioversion was performed. There was concern that not every QRS was being properly synchronized prior to the delivery of the first shock.

**Figure 2:**
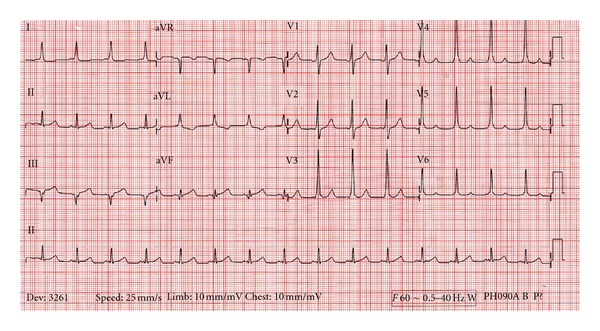
EKG showing sinus rhythm with underlying Wolff-Parkinson-White disease and classic upsloping QRS seen best in leads V3, V4, and V5.

The patient lapsed into ventricular fibrillation, and CPR was initiated. Endotracheal intubation was performed to protect the patient's airway. The patient was defibrillated at 200 J with return of pulses and normal sinus rhythm. The following ECG was obtained after successful cardioversion (see Figure 2).

The patient was admitted to the cardiac ICU, and the next day underwent ablation of his accessory pathway. He was successfully discharged on hospital day 3 in stable condition.

## 2. Wolff Parkinson White

The Wolff-Parkinson-White syndrome exists due to an accessory pathway between the atria and ventricles, allowing electrical impulses to bypass the AV node [1]. It should be considered in the differential of a young person with an initial presentation or prior history of supraventricular tachycardia, syncope, dizziness, shortness of breath, or palpitations [2]. Classic ECG findings (see Figure 2) include a delta wave from preexcitation of ventricular myocytes from conduction via the accessory pathway, followed by normal ventricular depolarization and a narrow QRS [1]. This accessory pathway can predispose the patient to one of several tachyarrhythmias, discussed below.

## 3. Rhythm Disturbances in Wolff Parkinson White

Patients with WPW can present with several forms of tachydysrhythmia, depending on the pathway of the aberrant rhythm [3].

1. Orthodromic AV reciprocating tachycardia [3]: this presents with a circus movement beginning with anterograde movement through the AV node, the ventricles, and the back through the accessory pathway. The appearance on ECG is a narrow complex tachycardia, typically with no clear delta wave.
2. Antidromic AV reciprocating tachycardia [3]: the impulses in this type of arrhythmia move down the accessory pathway, into the ventricles, and retrograde through the AV node; the ECG shows a regular, rapid, and wide-complex tachycardia which appears similar to monomorphic ventricular tachycardia.
3. Atrial fibrillation with RVR [4]: impulses are traveling down both the AV node and the accessory pathway. Due to nondecremental propagation through the accessory pathway, the ECG will show an irregular, rapid, and wide QRS tachycardia. One of the typical findings on ECG is variance in QRS complexes and RR intervals.

## 4. Case Discussion

Several interesting points come up from this case. First is that triage vital signs can be absent or erroneous. One may see either over- or undercounting the heart rate when pulse oximeters try to interpret unusual or rapid rhythms. For instance, a heart rate of 100 may be recorded as a rate of 200 (or 50). Cardiac monitoring, which is much more accurate, can avoid this pitfall. It additionally points to the essential nature of a focused clinical exam for heart sounds and peripheral or central pulses. This patient had a rate on ECG of nearly 300, but the patient's pulse was found to be an irregular, slow rate due to electromechanical dissociation and incomplete perfusion. An accurate and complete physical exam, coupled with cardiac monitoring, can give a clear understanding of the patient's state.

The correct treatment for tachydysrhythmias in the setting of WPW, whether known or suspected, is paramount to prevent decompensation.

In patients with a rapid narrow complex rate, consistent with orthodromic AV reciprocating tachycardia, treatment with adenosine is often effective if vagal maneuvers fail [5]. In these cases it is very difficult to recognize the delta wave, and the risk of using an AV nodal blocking agent is low. Other AV nodal blocking agents used in SVT may be used safely if adenosine therapy fails.

In patients with WPW who present with an antidromic AV-reciprocating tachycardia, the ECG is frequently indistinguishable form monomorphic ventricular tachycardia; keen physicians may identify P waves after the QRS [5]. The treatment is the same as for monomorphic V-tach, consistent with ACLS protocols for stable and unstable patients. The use of cardioversion and antiarrhythmic agents are safe.

In patients with WPW and atrial fibrillation, the most difficult problem is identifying the rhythm. Several other dysrhythmias can mimic this, including polymorphic ventricular tachycardia and atrial fibrillation with aberrant conduction from a bundle branch block [4]. Treatment in unstable patients with any of the aforementioned rhythms is synchronized cardioversion. Stable patients may be treated with procainamide or amiodarone. Patients should not be treated with beta blockers, calcium channel blockers, adenosine or digitalis glycosides; these run the risk of blocking AV nodal conduction and redirecting conduction through the accessory pathway resulting in a more rapid, unstable tachycardia [1]. Heart rates greater than 300 can be seen, which can predispose to ventricular fibrillation or ventricular tachycardia.

Cardioversion, which was attempted in the case above, presents both risks and benefits in patients with WPW who have rapid rates. In this case, it is possible that the incomplete syncing led to an R-on-T phenomena, stimulating incompletely depolarized tissue and triggering ventricular fibrillation. If performing sequential cardioversion, assure that syncing is enabled before each delivered shock, as some devices will deactivate the sync function after a shock is delivered. Caution must be taken to avoid AV nodal blocking agents and perform a synced cardioversion in patients with WPW that present in atrial fibrillation with rapid ventricular response.

It is an early teaching point in medical training to beware AV nodal blocking agents in WPW. As clinicians gain experience, they should review the reasoning behind this and learn to recognize history, physical exam, and ECG findings that suggest WPW as an underlying etiology. Treating the most common tachydysrhythmias in WPW is similar to treating SVT and V-tach; if there are no indications for immediate synced cardioversion, review the ECG and history and choose an antiarrhythmic carefully or pursue elective cardioversion.
